# The Role of Nerves in Skeletal Development, Adaptation, and Aging

**DOI:** 10.3389/fendo.2020.00646

**Published:** 2020-09-23

**Authors:** Ryan E. Tomlinson, Blaine A. Christiansen, Adrienne A. Giannone, Damian C. Genetos

**Affiliations:** ^1^Department of Orthopaedic Surgery, Thomas Jefferson University, Philadelphia, PA, United States; ^2^Department of Orthopaedic Surgery, School of Medicine, University of California, Davis, Sacramento, CA, United States; ^3^Department of Anatomy, Physiology, and Cell Biology, School of Veterinary Medicine, University of California, Davis, Davis, CA, United States

**Keywords:** mechanotransduction, nervous system, bone, skeleton, aging, disuse

## Abstract

The skeleton is well-innervated, but only recently have the functions of this complex network in bone started to become known. Although our knowledge of skeletal sensory and sympathetic innervation is incomplete, including the specific locations and subtypes of nerves in bone, we are now able to reconcile early studies utilizing denervation models with recent work dissecting the molecular signaling between bone and nerve. In total, sensory innervation functions in bone much as it does elsewhere in the body—to sense and respond to stimuli, including mechanical loading. Similarly, sympathetic nerves regulate autonomic functions related to bone, including homeostatic remodeling and vascular tone. However, more study is required to translate our current knowledge of bone-nerve crosstalk to novel therapeutic strategies that can be effectively utilized to combat skeletal diseases, disorders of low bone mass, and age-related decreases in bone quality.

## Introduction

The presence and purpose of nerves in bones has been under investigation for many decades, beginning in earnest with the use of routine histological preparations and electron microscopy in the 1960s, 1970s, and 1980s (1–3). These studies were motivated by the desire for greater understanding of skeletal pain, such as that induced by surgical operations to resect tumors or stabilize broken bones. Many early studies using denervation models to abruptly sever the nerve supply of bones reported minimal effects of diminished nerve activity on bone mass or accrual in a variety of animal species. Nonetheless, recent immunohistochemical studies have revealed abundant sensory, sympathetic, and parasympathetic axons of the peripheral nervous system that terminate in bone (4–10). As a result, research into each of these nerve populations has revealed the unique functions of each subtype within the skeletal microenvironment. However, much remains to be uncovered. In this review, we will discuss the sensory, sympathetic, and parasympathetic actions on bone, as well as the current understanding of nerve roles during skeletal development, adaptation to mechanical load, and aging.

## Skeletal Innervation

### Sensory Nerves in Bone

The somatic nervous system (SNS) includes the sensory nerves distributed throughout the body after their extension from the dorsal root ganglia during development. Skin is well-innervated by sensory nerves, along with the underlying bone, joints, tendon, and muscle. These nerves serve a variety of important roles in the body, including the production of signals that provide spatial orientation (proprioception), interpret pain and noxious stimuli (nociception), recognize temperature changes, and allow the perception of non-painful tactile stimuli (11–14). Most sensory nerves can be categorized by their expression of channels and receptors (15); these include toll-like receptors (TLRs), transient receptor potential (TRP) ion channels, and receptor tyrosine kinases (RTKs) as well as the recently identified mechanosensitive Piezo channels (15, 16). These receptors are utilized to initiate the appropriate intracellular signaling as well as the neuropeptide or neurotransmitter release. In bone, nearly all of the thinly myelinated and unmyelinated sensory nerves express neurotrophic receptor tyrosine kinase type 1 (TrkA), the high affinity receptor for nerve growth factor (NGF) (10, 17). This specialization is likely due to the NGF expression that occurs during the initiation of primary and secondary ossification in endochondral bone formation (18, 19). Nonetheless, innervation of bone is most dense in the periosteum and marrow spaces, with relatively few nerves present in the mineralized bone (9, 10). Furthermore, innervation density is increased at sites nearest to active bone remodeling surfaces (20).

### Function of Sensory Nerves in Skeletal Pain

One of the original motivations for studying sensory nerves in bone was to determine the mechanisms of osseous pain. This objective has been bolstered by the prevalence of musculoskeletal pain, including lower back pain, joint pain, and fracture pain, which collectively are the leading cause of disability in the world (21). Much of this work has been centered on NGF, which is expressed by osteoblasts and acts directly on sensory nerve axons present in bone through TrkA receptors to induce skeletal pain; furthermore, NGF also functions to enhance the activation of other nociceptive pathways in skeletal sensory nerves (22). As a result, the blockade of NGF activity has been explored extensively in a variety of animal models of skeletal pain. For example, anti-NGF antibodies profoundly reduce osteosarcoma-related bone pain in mice as well as tumor-induced nerve sprouting in a preclinical model of metastatic prostate cancer (23, 24). Furthermore, consistent with the wide-spread expression of NGF observed in fracture (25, 26), anti-NGF antibodies decrease fracture-related pain behavior in mice (27, 28). Subsequent research suggested that analgesia using anti-NGF antibodies can be achieved without affecting fracture healing outcomes (29), although others have shown that silencing the activation of TrkA diminishes innervation and stress fracture healing in mice (30). This concept of NGF acting as an osteoanabolic agent is consistent with earlier work, which reported that topical application of NGF to rib fractures decreased healing time in rats (27) and improved healing outcomes in distraction osteogenesis in rabbits (28). Nonetheless, a complete understanding of the role of NGF-TrkA signaling in bone healing should be a research priority, since the humanized monoclonal anti-NGF antibody Tanezumab (Pfizer and Lilly) received FDA Fast Track approval in 2017 as the first in a new class of non-opioid pain relievers. This approval followed a halt in Phase III clinical trials in 2010 due to an increased incidence of adverse skeletal events, which remains incompletely understood (31).

### Release of Neuropeptides by Skeletal Sensory Nerves

Stimulation of sensory nerves in bone may result in the release of neuropeptides, particularly calcitonin gene-related peptide (CGRP) and substance P (SP), but may also include glutamate and pituitary adenylate cyclase-activating polypeptide (PACAP) (32). A potential role for neuropeptides to act as bone therapeutics has been investigated extensively, since both osteoblasts and osteoclasts express the necessary receptors to for direct cell-autonomous activation (33, 34). In general, CGRP increases osteoblast bone formation through stimulation of Wnt signaling and inhibition of apoptosis (35, 36). Furthermore, CGRP appears to inhibit osteoclast differentiation and function (37, 38). Consistent with these findings, mice lacking αCGRP have low bone mass as a result of decreased bone formation (39). SP appears to increase bone resorption as well as bone formation, although its contribution toward formation outweigh its role in bone resorption and lead to impaired material and structural bone strength (40, 41). These findings are consistent with results from previous *in vitro* experimentation that have demonstrated both mechanisms (42–44). Neuropeptide release within the skeleton is potentiated by osteoblast-derived NGF, which increases both basal and stimulus-evoked release of SP and CGRP from spinal cord slices *in vitro* (33). Nonetheless, the therapeutic application of these osteoanabolic neuropeptides toward diseases of low bone mass may be limited by drug delivery, since neuropeptides are widely active outside of bone.

### Autonomic Nervous System (ANS) in Bone

The peripheral nervous system also includes the autonomic nervous system (ANS), which is further divided into the sympathetic and parasympathetic nervous systems. In general, the action of these two systems oppose each other and serve to coordinate unconscious activities of the body, such as breathing and blood pressure regulation. Coordinated action of these opposing systems involves unique signaling mechanisms: sympathetic nerves release of norepinephrine to activate α- and β-adrenergic receptors, whereas parasympathetic nerves release acetylcholine to activate muscarinic acetylcholine receptors (mAChR) and nicotinic acetylcholine receptors (nAChR). Both sympathetic and parasympathetic nerves have been identified in the bone, and are typically observed in close contact with large vascular structures in the long bones (5, 7, 34). Tyrosine hydroxylase (TH), the rate limiting enzyme in the synthesis of catecholamines, is typically used as an immunohistochemical marker for sympathetic nerves. TH^+^ axons are typically observed with a spiral morphology in the bone marrow, essentially wrapping around blood vessels (10, 45). Conversely, axons expressing vesicular acetylcholine transporter (VAChT) and choline aceltyltransferase (ChAT), two markers for parasympathetic nerves, can also be readily observed in the marrow space of long bones (46). The innervation density of these nerve axons is less well-described than sensory nerves, but is presumed to follow a similar pattern.

### Function of ANS in Bone

The major function of the ANS on the skeleton is restraint of bone remodeling (37). Specifically, activation of the sympathetic nervous system acts to stimulate bone resorption as well as negatively affect bone formation (37, 38). Conversely, the parasympathetic nervous system activity inhibits bone resorption, which results in bone mass accrual (46). These activities may also be related to the general circadian rhythm of the autonomic nervous system. Sympathetic nervous activity is generally dominant during the day, which is the peak time for bone resorption, while parasympathetic nervous activity is generally dominant at night, when bone formation peaks (47–49). Osteoblasts and osteoclasts express a wide variety of adrenergic receptors that could be activated in response to norepinephrine released from sympathetic nerve terminals (50, 51). Similarly, osteoblast and osteoclasts may be able to respond to the release of acetylcholine from parasympathetic nerve terminals due to their expression of the α2 and β2 subunits of the nAChRs; mAChRs expression is absent in these cell types (46). Due to the expression of these receptors on bone cells, it has been presumed that the ANS exerts its effect on the skeleton through the release of neurotransmitters in close proximity to bone cells, which subsequently bind to their cognate receptors to initiate a biological response. However, recent work reporting relatively limited direct interaction of ANS nerve fibers with skeletal cells suggests that an alternative diffusion-based mechanism may be plausible (32, 52). Nonetheless, mice lacking β2 adrenergic receptor in the osteoblast lineage have increased bone mass in adulthood, due to increased bone formation and decreased bone resorption (50). This encouraging result, along with the known safety profile of “β blockers” (β adrenergic antagonists), suggested that pharmacological blockade of sympathetic nervous signaling would increase bone mass and decrease fracture risk in humans. Surprisingly, subsequent preclinical research utilizing β adrenergic receptor agonist (salbutamol) or antagonist (isoprenaline) failed to recapitulate the previous findings in mice; instead, these drugs were both associated with bone loss, mostly due to increased bone resorption (53, 54). Similarly, a randomized clinical trial observed no significant effects of either β2 adrenergic agonists or antagonists on bone turnover in adults (55). A meta-analysis of 16 studies published in 2014 reported that the use of β blockers decreased overall fracture risk by 15%, with β1-specific blockers most strongly associated with the reduction in risk (56). Consistent with this report, a recent randomized controlled trial utilized the relative selectivity of β-blockers to show that patients treated with β1-selective drugs had improved parameters of bone density and turnover (57). In total, much of the direct and specific effects of the autonomic nervous system, as well as the potential therapeutic opportunities in modulating this signaling pathway, remains to be determined.

### Release of Neuropeptides by ANS in Bone

Similar to sensory nerve axons of the SNS, sympathetic nerves of the ANS can also release neuropeptides, particularly in response to stress. One such neurotransmitter released by sympathetic nerves is neuropeptide Y (NPY), which can signal through one of five NPY receptors that are expressed in both the central and peripheral nervous systems (58, 59). A role for NPY in bone homeostasis was first recognized in 2002, when Y2 receptor null mice were found to have significant increased trabecular bone volume due to increased osteoblastic activity without an alteration in osteoclast resorptive area (60). Similarly, in the setting of ovariectomy, mice lacking hypothalamic Y2 receptors were found to be protected from bone loss through an increase in osteoblastic activity (61). More recently, mice in which NPY was expressed exclusively in noradrenergic nerves of mice otherwise lacking NPY were used to demonstrate that NPY acts both centrally and peripherally through Y2 receptors to protect against stress-induced loss of bone mass (62). Consistent with these studies, *in vitro* work utilizing primary osteoblasts has revealed a direct inhibitory effect of NPY on osteoblast differentiation, indicating NPY exerts its effects both directly and indirectly on bone (63–65). Similarly, nerve axons expressing vasoactive intestinal peptide (VIP) have been observed in bone for some time (4, 66). Recent interest in this neuropeptide secreted from the ANS has shown that it promotes osteogenic differentiation *in vitro* and stimulates bone repair when delivered *in vivo* (67). Alternately, VIP appears to be implicated in the progression of osteoarthritis through actions on subchondral bone sclerosis and vascularity (68).

### Modifiers of Sympathetic Signaling in Bone

Restraint of sympathetic signaling on bone is achieved *via* antagonistic sympathetic projections and degradation or sequestration of sympathetic neurotransmitters; each are implicated in an aging skeletal phenotype. Endocannabinoids, such as 2-arachidonylglycerol (2-AG), are generated by bone cells and act on CB1 receptors on skeletal sympathetic nerve endings. In support of endocannabinoid restraining the inhibitory effect of sympathetic transmission of skeletal mass and microarchitecture, global deletion of the CB1 receptor (*Cnr1*) produces a skeletal phenotype characterized by decreased trabecular microarchitecture, low bone mass, and increased osteoclast activation (69). However, functional impact of cannabinoid receptor signaling on restraint of SNS outflow and resultant skeletal effects are clouded by contrasting results from different groups, related to choice of mouse models, sex, and animal age. For example, enhanced bone mass and resistance to ovariectomy-induced bone loss was recently reported in congenic Swiss albino ABH and CD1 congenic *Cnr1*-deficient mice (70). However, divergent skeletal phenotypes were observed in C57BL/6J vs. CD1 *Cnr1*-deficient mice producing both loss and gain, respectively, of bone mass in the absence of *Cnr1*. Furthermore, the divergent skeletal phenotype was sexually-dimorphic in CD1 *Cnr1*^−/−^ mice, affecting males but not females, whereas the effect was independent of sex in C57BL/6J strain of mice (69).

Neurotransmitter clearance from the synaptic cleft presents as another mechanism to influence magnitude or duration of sympathetic signaling on the skeleton. Clearance of NE from the synaptic cleft by the norepinephrine transport NET (*SLC6A2*), a sodium- and chloride-dependent monoamine transporter. Provided that sympathetic signaling exerts negative skeletal effects *via* RANKL-mediated osteoclast activation and inhibition of osteoblast function (71), pharmacologic inhibition or genetic deletion of NET would be expected to produce a high bone mass phenotype. Whereas, mature osteoblasts express high levels of NET, its inhibition with reboxetine elicited sexually-dimorphic reductions in osteoblast number and bone mineralization in male, but not female, mice, findings which were also recapitulated in global *Slc6a2*^−/^^−^ mice. Clearance of NE may contribute to aging-associated skeletal wasting, as NE uptake is greater in young (3 months) than aged (18 months) mice, reflective from decreased *Slc6a2* expression increased tibial NE content with age (71). These results demonstrate that NE clearance and catabolism is a fundamental aspect of skeletal homeostasis with a potential function in involutional bone loss, yet the unexpected results from pharmacologic inhibition or genetic deletion of *Slc6a2*^−/−^ reveal the need for inducible murine knockout models to more clearly detail where and when loss of *Slc6a2* or *Cnr1* most potently influence bone mass.

## Nerves in the Developing Skeleton

### Timing of Skeletal Innervation During Endochondral Ossification

**Figure 1:**
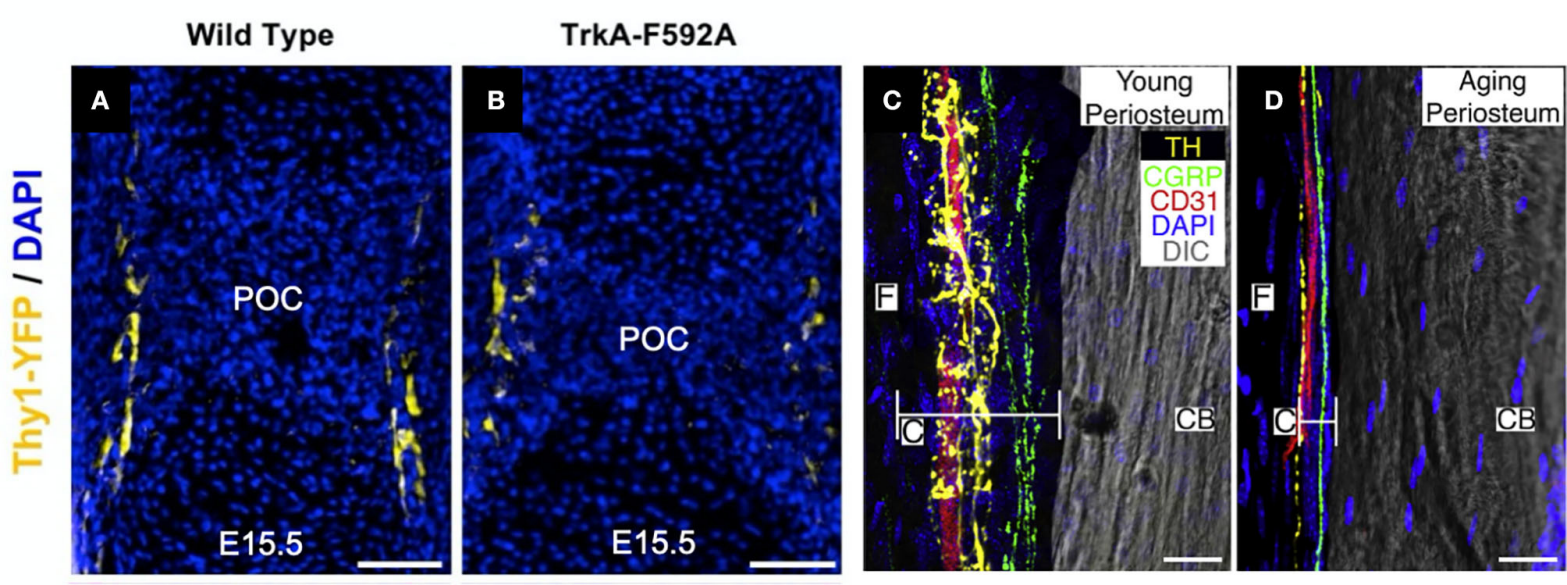
Nerves in developing, young, and aging bone. **(A)** Thy1-YFP reporter mice were used to visualize nerve axons in the perichondrial region near the primary ossification center (POC) at embryonic day 15.5. **(B)** Inhibition of NGF-TrkA signaling using TrkA-F592A mice diminished the density of nerve axons in this region. Scale bars are 100 μm [adapted from (19)]. **(C)** Utilizing a 120 μm confocal *z*-stack, CD31^+^ blood vessels (red), CGRP^+^ sensory nerve axons (green), and TH^+^ sympathetic nerve axons (yellow) can be readily visualized in the periosteum of 10-day-old (young) mice. **(D)** In mice 24 months of age (old), sensory and sympathetic nerve fibers as well as blood vessels remain intact but markedly diminished in the thinner periosteum. Cambium (C) and fibrous (F) layers of the periosteum and cortical bone (CB) are labeled. Scale bars are 15 μm [adapted from (74)].

The exact location, timing, and subtype of nerves entering developing bone has been a topic of research interest for some time. Early work established that the innervation of bone occurs approximately simultaneously with endochondral ossification during embryonic development, including studies in mice illustrating a functional nerve supply in areas of high osteogenic activity by embryonic day 15 as well as the presence of CGRP immunoreactive nerves by embryonic day 16.5 (72, 73). Consistent with these studies, we have recently demonstrated that TrkA expressing sensory nerves arrive at the perichondrial surface of developing bone at embryonic day 14.5 in mice (Figure 1A), in response to the expression of the neurotrophin NGF by osteoprogenitors and coincident with the initiation of primary ossification (75). After birth, nerve density in bone continues to increase, coinciding with the bone modeling and remodeling necessary for shaping long bones. Sensory nerve axons expressing CGRP and Substance P are present at postnatal day 1 in the epiphysis and endosteum of the distal femur and proximal tibia, and by postnatal day 6, these sensory nerves appear in the cartilage canal and 2 days later in the secondary ossification centers (76). Similar to the invasion of the primary ossification center by sensory nerves, the sensory nerve axons entering the secondary ossification center through cartilage canals is in response to the expression of NGF at the epiphysis and the majority of these nerves express TrkA (75). Unlike sensory nerves, autonomic fibers staining for NPY do not appear in bone until postnatal day 4 (72). The autonomic fibers first appear as single, non-vascular, branching fibers in the tibial and femoral periosteum. NPY fibers next appear in the medullary cavity accompanying blood vessels until postnatal day 14, when the occurrence of the fibers decreases in all bone compartments.

### Effects of Diminished Skeletal Innervation on Developing Bone

Although the presence of nerves during primary and secondary ossification is well-documented, their function during skeletal development remains poorly understood. Denervation and associated models of nerve inactivation provide some insights into the role of peripheral nerves during bone development. For example, sectioning the sciatic nerve in 1-month-old rats reduced metatarsal length 3–5%, while femora and tibiae, containing femoral and obturator nerves which could potentially compensate for sciatic neurectomy, were unaffected (77). Notably in this study, early reductions in bone length were maintained, and were not exacerbated, up to the end of the 12-week study. Similarly, sciatic neurectomy prevented gains in bone mass and improvements in microarchitecture, instead inducing considerable trabecular bone loss in growing rats, due to decreased bone formation and increased bone resorption, though it is unclear how much of these bone changes were due to disuse (78). In mice either globally- or neuronally-deficient in semaphorin 3A, an axonal chemorepellant important to axon guidance, decreases in sensory innervation of trabecular bone reduced bone mass *via* decreased bone formation in 8-week old mice (79). Mice lacking TRPV1 (capsaicin receptor/vanilloid receptor1), a cation channel involved in nociception found on sensory nerves, displayed a similar phenotype to wildtype mice, with similar size and bodyweight. However, TRPV1 knockout mice exhibited a reduction in the basal levels of the osteoclast activation biomarker TRAP in the femur (80) and ovariectomy of these knockout mice did not cause the elevation in TRAP levels or bone loss as normally occurs in wildtype mice. αCGRP knockout mice displayed osteopenia associated with low bone formation rate without changes in osteoblast number or surface (39). In our previous study (81), we investigated bone development in mice treated with capsaicin as neonates to destroy unmyelinated and small diameter myelinated sensory neurons (82, 83). We found that neonatal capsaicin treatment in mice modestly decreased femur length, femur cross-sectional area, and trabecular bone thickness, but did not reduce mechanical properties or bone remodeling rates. In another study, we showed that nerve growth factor (NGF) signaling through neurotrophic tyrosine kinase receptor type 1 (TrkA) directs sensory innervation during long bone development to promote vascularization and osteoprogenitor differentiation (75). Inactivation of NGF or TrkA signaling during embryogenesis in mice impaired sensory innervation (Figure 1B), delayed vascularization of ossification centers, decreased numbers of osteoprogenitors, and decreased femoral length and volume. In total, these studies indicate that sensory innervation is required for attaining normal bone mass and length, as well as vascularization, during skeletal development. Future work should determine if any specific osteogenic factors are delivered by sensory nerves to bone.

## Nerves and Skeletal Adaptation

Bone tissue contains a dense network of sensory and sympathetic nerve fibers, which appears to play important roles in bone modeling, remodeling, metabolism, and adaptation (84). For example, in a study of bone remodeling induced by maxillary molar removal in rats, investigators found that normal tibial growth was not impaired by neonatal sympathectomy (guanethidine treatment) or sensory denervation (capsaicin treatment), but that osteoclast surface was increased 45% in sympathectomized animals and decreased 21% in sensory denervated animals (85). These data indicate that both sympathetic and sensory nerves play a role in bone adaptation, and that these unique fiber types may play opposing roles on skeletal adaptation. We have provided a concise summary of the previous work that studied the roles of sensory and sympathetic nerves in scenarios of increased (Table 1) and decreased (Table 2) mechanical loading.

**Table 1:** Increased mechanical loading and altered nerve function.

	**Model of altered nerve function**	**Loading method**	**Effect on bone**	**References**
Sensory nerves	**↓**Sensory function—perineural anesthesia of brachial plexus with bupivacaine in rats	Ulnar compression	**↓**Labeled bone area	(80)
	**↓**Sensory function—perineural anesthesia of brachial plexus with bupivacaine in rats	Ulnar compression	**↓**Labeled bone area	(82)
	**↓**Sensory function—inhibition of TrkA signaling by 1NMPP1 in mice	Ulnar compression	**↓**Bone formation rate **↓**Wnt/β-catenin activity in osteocytes **↓**Periosteal nerve sprouting	(19)
	**↑**Sensory function—exogenous NGF administration in mice	Ulnar compression	**↑**Bone formation rate **↑**Wnt/β-catenin activity in osteocytes	(19)
	**↓**Sensory function—neonatal capsaicin treatment in mice	Tibial compression	**↑**Bone mineral content **↑**Mineral apposition rate	(84)
Sympathetic nerves	**↓**Sympathetic function—guanethidine sulfate or propranolol treatment in mice	Tibial compression	No effect	(83)
	**↓**Sympathetic function—propranolol treatment in mice	Tibial compression	No effect	(85)
	**↓**Sympathetic function—propranolol treatment in ovariectomized rats	Treadmill exercise	**↓**Trabecular BV/TV and Tb.Th	(86)
	**↑**Sympathetic function—salbutamol treatment in rats	Treadmill exercise	**↓**MAR, Tb.Th, ultimate force, stiffness, Young's modulus	(53)
	**↑**Sympathetic function—salbutamol treatment in ovariectomized rats	Treadmill exercise	**↓**Trabecular BV/TV and Tb.Th	(87)
	**↓**Sympathetic function—genetic deletion of β1-adrenergic receptors and/or β2-adrenergic receptors in mice	Tibial compression	**↓**BMD, Tb.Th, MAR, BFR/BS in Adrb1^−/−^ and Adrb1b2^−/−^ mice	(88)

**Table 2:** Decreased mechanical loading and altered nerve function.

	**Model of altered nerve function**	**Loading method**	**Effect on bone**	**References**
Sensory nerves	**↓**Sensory function—neonatal capsaicin treatment in rats	Molar extraction	**↓**Osteoclast surface	(78)
	**↓**Sensory function—capsaicin treatment in adult rats	Hindlimb unloading	**↑**Energy to failure	(92)
Sympathetic nerves	**↓**Sympathetic function—guanethidine treatment in rats	Molar extraction	**↑**Osteoclast surface	(78)
	**↓**Sympathetic function—guanethidine or propranolol treatment in mice	Hindlimb unloading	**↑**Trabecular BV/TV **↑**MAR, MS/BS, BFR/BS **↓**Oc.N and Oc.S	(93)
	**↑**Sympathetic function—isoproterenol treatment in mice	Hindlimb unloading	No effect	(93)
	**↓**Sympathetic function—guanethidine sulfate or propranolol treatment in mice	Sciatic neurectomy	No effect	(85)
	**↓**Sympathetic function—propranolol treatment in rats	Hindlimb unloading	**↑**Trabecular Bone Volume **↑**MAR, BFR/BS **↓**Resorbing surface	(94)
	**↑**Sympathetic function—dobutamine treatment in rats	Hindlimb unloading	**↑**BMD, BMC, Bone Area **↑**MAR, MS/BS, BFR/BS	(95)
	**↑**Sympathetic function—dobutamine treatment in rats	Hindlimb unloading	**↑**BV/TV, Tb.Th, Tb.N **↑**OS/BS, Ob.S/BS **↑** MAR, MS/BS, BFR/BS **↓** Osteocyte apoptosis	(96)

### Peripheral Nerves Support Load-Induced Bone Formation

The role of peripheral nerves in sensing and responding to mechanical stimuli is an area of equal parts interest and contradiction. Early studies reported that denervation had essentially no effect on the bone formation response to mechanical loading. For example, intermittent loading (bending) initiated similar magnitudes of cortical bone formation in the denervated rabbit tibia as in intact tibias (86). This led the authors to conclude that the nervous system has no significant effect on the functional adaptation of bone. However, recent studies have established a notable role of peripheral nerves in bone mechanosensing and adaptation to mechanical stimuli. A pivotal study used bupivacaine to induce perineural anesthesia of the brachial plexus of rats to achieve temporary neuronal blocking prior to ulnar compression (87). They found that temporarily blocking neuronal signaling reduced bone formation (total labeled bone area) by 81% in the compressed ulna relative to sensory intact ulnae. Further studies by this group revealed that mechanical loading increased bone formation in the contralateral limb and at other non-loaded skeletal sites, which was modulated through sensory nerves (88, 89); however, load-induced increases in contralateral bone formation have been directly contradicted by others (90) and indirectly by the large number of related studies that utilize contralateral limbs as an internal control.

Our study investigating bone adaptation to increased mechanical loading in mice treated with capsaicin to induce destruction of TRPV1-expression peripheral nerves found that tibial compression increased cortical bone area in the loaded tibia, accompanied by changes in bone formation, which was generally greater in capsaicin-treated mice than in vehicle-treated mice (91). In contrast, our study of NGF-TrkA signaling in sensory nerves in bone showed that elimination of TrkA signaling attenuated bone formation and reduced Wnt/β-catenin activity in osteocytes in bones loaded by axial forelimb compression. Furthermore, administration of exogenous NGF to wild-type mice significantly increased load-induced bone formation and Wnt/β-catenin activity in osteocytes (75). The contrasting results from these two studies of decreased sensory nerve signaling in bone suggest a heterogenous population of sensory nerves in bone with non-overlapping functions in strain adaptive bone remodeling.

The role of the sympathetic nervous system in the anabolic bone response to mechanical loading is unclear. One study in mice reported that sciatic neurectomy enhanced tibial compression-induced cortical bone formation, but pharmacological blockade of the SNS with guanethidine sulfate or propranolol did not affect the bone formation response (90). The same group found that load-induced bone formation and unloading-induced bone resorption were unaffected by propranolol or guanethidine sulfate treatment (92). Another study found that either propranolol treatment or exercise in ovariectomized rats was able to partially preserve trabecular bone volume, but these treatments did not have a synergistic effect, and in fact exhibited an antagonist effect on trabecular bone (93). Similarly, treatment of rats with a selective β2-adrenergic receptor agonist (salbutamol) decreased bone mineral density and increased bone resorption, and salbutamol treatment mitigated the beneficial effects of treadmill exercise on bone structure in these rats (53, 94). In genetic mouse models of β1-adrenergic receptor and/or β2-adrenergic receptor deficiency, tibial compression induced increases in bone density, trabecular and cortical microarchitecture, and bone formation in *Adrb2*^−/^^−^ and wild-type mice, but not in *Adrb1*^−/^^−^ or *Adrb1b2*^−/^^−^ mice, suggesting that β1, but not β2, has a role in mechanoadaptation to mechanical stimulation (95).

### Peripheral Nerve Impact in Disuse

The initial rapid loss of bone following spinal cord injury suggests that factors other than disuse osteoporosis may drive the catabolic skeletal response (96). Indeed, electrical stimulation of muscle does not restore bone mass *post* trauma (97). Similarly, unilateral sciatic nerve transection causes bone loss not only in the denervated limb, but in the contralateral limb as well—even when use remains unchanged (98). Altogether, these data suggest that peripheral nerve activity may modulate the bone resorption response during disuse. A study of hindlimb unloading-induced bone loss in capsaicin-treated rats showed that both capsaicin treatment and 4 weeks of hindlimb unloading resulted in considerable loss of trabecular bone at the proximal tibia, but that hindlimb unloading of capsaicin-treated rats did not promote further bone loss (99). Altogether, these data suggest that diminished sensory nerve function diminishes bone volume but may make the bone less sensitive to the mechanical environment. Together, these data suggest that diminished sensory nerve function diminishes bone volume and may make the bone less sensitive to the mechanical environment. Moreover, results from a model of disuse-induced remodeling in the mandible is consistent with this view. Here, significantly decreased osteoclast surface was observed in rats with decreased sensory nerve function due to neonatal capsaicin treatment (85).

Sympathetic nerves have a notable effect modulating unloading-induced bone loss. One study reported that inhibiting sympathetic nerves in mice using propranolol or guanethidine suppressed bone loss associated with hindlimb unloading by diminishing the reduction in osteoblast activity and the increase in osteoclast activity associated with unloading (100). Conversely, activating sympathetic nerves using isoproterenol reduced bone mass in normally loaded mice, but did not cause additional bone loss in hindlimb unloaded mice. Similarly, others found that treatment of rats with propranolol or a leptin analog during 28 days of hindlimb unloading reduced unloading-associated bone loss; propranolol treatment effectively preserved bone formation and prevented increased bone resorption, while leptin analog treatment was only able to prevent changes in osteoclastic bone resorption (101). Conversely, treatment of rats with a β1-adrenergic receptor agonist (dobutamine) attenuated hindlimb unloading-induced bone loss, prevented the decline in bone formation induced by unloading, and diminished unloading-induced osteocyte apoptosis (102, 103).

### Load-Induced Neurotransmitter Expression in Bone

The first evidence of mechanical regulation of neurotransmitters was the observation that ulnar loading in rats decreased expression of GLAST, a glutamate/aspartate transporter previously thought to be present only in mammalian CNS (104). Quantification of CGRP, VIP, and SP in the rat ulna after mechanical loading (ulnar compression) using ELISA revealed that CGRP concentrations in both the loaded and contralateral limbs were reduced 1 h after loading, and that this reduction was sustained for at least 10 days (87). Bilateral decreases in SP concentrations were also observed, although the effect was less persistent. Ulnar VIP concentrations were increased bilaterally 10 days after mechanical loading at medium or high strain magnitudes. In contrast, both CGRP and SP levels were increased in the sciatic nerve after 4 weeks of cast immobilization (105). Our results partially agreed with these data, as mechanically loading tibias in mice resulted in significantly decreased SP concentrations, but increased CGRP concentrations relative to controls, while unloaded tibias exhibited trends toward increased concentrations of both CGRP and SP (91).

## Nerves and the Aging Skeleton

Chronological aging causes cell and tissue dysfunction, which compromises individual capacity to maintain homeostasis. In the context of the skeleton, uncoupled remodeling promotes net bone loss, characterized by reductions in bone mineral density and bone strength and increased fracture risk. Suggestive links between autonomic tone and bone strength with aging are evident in associations such as increased sympathetic tone in post-menopausal women (106) who are at risk of osteoporotic fractures, and hereditary neuropathies with skeletal manifestations [reviewed in (32)]. Provided the distribution and patterning of sympathetic and sensory nerves in bone, changes in fiber presentation, function, or restraint have each been presented as correlative—if not causative—for bone loss with age.

### Fiber Number and Density

Aging reduces nerve fiber frequency and their organization. A recent study evaluated sensory and sympathetic innervation of the periosteum, cortical bone, and bone marrow in femora of C57BL/6 mice at 10 days, 3 months, or 24 months of age (74). They observed highest density of sensory (CGRP^+^) and sympathetic (TH^+^) neurons in the inner cambial layer of the periosteum; CGRP^+^ sensory fibers displayed a linear pattern along the long axis of the femur, whereas TH^+^ sympathetic fibers were highly branched and closely associated with CD31^+^ blood vessels (Figure 1C). Despite substantial periosteal thinning with age—~75% reduction in total thickness, with greatest reduction in the cambium (~90% decrease)—which reduced total fiber number, fiber density was greatest in aged animals, likely owing to the dramatic reductions in periosteal thickness in which fibers were located (Figure 1D). Within cortical bone, CGRP^+^ and TH^+^ fibers were observed exclusively in Haversian canals, and fiber morphology was similar as observed in the periosteum. TH^+^ fibers decreased in aged animals (~48–14%), whereas a similar fraction (26%) of fibers were CGRP^+^ in adult and aged animals, and there was a modest reduction in CD31^+^ blood vessels (89% in 3 months vs. 62% in 24 months). There were no statistically distinct differences in fiber density in bone marrow as a function of chronological age. However, other studies do not fully corroborate these findings. Using a similar approach in a rat model, reductions in cambium thickness with age were observed without change in fibrous periosteum thickness or periosteal innervation (107); in a human study of femora and tibiae of aged individuals (68–99 years), significant intraindividual differences in periosteal thickness of tibia and femur were reported, yet there was no correlation of periosteal thickness of either bone as a function of age or weight (108). Thus, whereas supportive evidence for decreased periosteal thickness as a consequence of aging suggests a relationship to decreased nerve fiber number and/or density, more detailed investigation across a number of species is necessary to faithfully support such conclusions.

Alterations in fiber density with aging may drive reduced tissue function, specifically aging of the hematopoietic stem cell niche within the skeleton. By comparing young (8–10 weeks) and old (20–24-month-old) mice on a Nestin-GFP reporter background, aging associated with remodeling of bone marrow vascular architecture: total vascular density (CD31^+^ CD144^+^) increased in aged mice which was driven by reductions in arteriolar segment length despite cellular expansion away from the endosteum and which converged on the central vein (109). Concomitant with arteriolar remodeling were reductions in TH^+^ fiber density, total nerve density (β-III tubulin), and neural dysfunction (reduced synaptophysin density as a marker of synaptic contacts between blood vessels and nerves). Surgical transection of femoral and sciatic nerves in young mice fully recapitulated the effect of age, indicated by absence of TH^+^ fibers, expanded myeloid-biased CD41^+^ HSCs with reduced competitive engraftment potential, and vascular remodeling. Niche-derived noradrenaline maintains HSC function, as β2- or β3-selective sympathomimetics reduced both absolute numbers and frequencies of MSCs and ECs in aged mice, comparable to young mice; further, β3 agonists increased donor HSC engraftment following transplantation in aged mice and rescued the premature aging phenotype in denervated mice. Conversely, constitutive deletion of *Adrb3* accelerated HSC niche aging in young mice. Thus, age-associated alterations in bone marrow innervation and vasculature drive hallmarks of immune dysfunction, although the direct impact on skeletal involution requires elaboration.

### Sympathetic Outflow, Aging, and Skeletal Disease

Restraint of sympathetic outflow in the skeleton presents as another potential mechanism whereby age-associated impairment of cell function produces organ-level dysfunction. For example, sustained presentation of NE within the skeleton—given its established catabolic effect on the skeleton—may drive imbalanced remodeling as observed in older animals and humans. Indeed, differential clearance of NE by the norepinephrine transporter NET (*Slc6a2*) was observed to be a function of age: specific NE uptake from flushed femoral cortical bone was greater in young (3 months) than aged (18 months) mice (71). Correspondingly, basal NE content was greater in aged compared to young mice, although this did not associate with increased sympathetic outflow in older animals. Thus, inadequate clearance of NE in aged bone may contribute to skeletal wasting due to sustained β2 adrenergic stimulation. Similarly, the cannabinoid receptors Cb1 (*Cnr1*) and CB2 (*Cnr2*), which restrain sympathetic signaling, are implicated in age-related bone loss and joint disease. Dual deletion of both Cb1 and CB2 (*Cnr1*^−/^^−^/*Cnr2*^−/^^−^*)* mice reveal attenuated bone loss as a function of age or estrogen status resulting from deficits in osteoclast formation (110). Further, CB receptor agonists protect against both collagen- (111, 112) and destabilization-induced arthritis (113), and loss of *Cnr2* delays osteoarthritis progression (113). Whilst illuminating, such studies do not identify which cells mediate the observed influence: they do not reveal if cell-autonomous defects in osteoclastogenesis *in vivo* mitigate bone loss, nor do they establish sympathetic involvement.

### Neurotrophin Presentation With Aging

Despite a name suggesting neural-specific expression and function, neurotrophins and their receptors are highly expressed in osteochondrogenic cells during development and repair [reviewed in (114)] Thus, changes in neurotrophin ligand or receptor expression in the skeleton can alter the ingrowth or maintenance of neural fibers in the skeleton. Whether aging influences presentation of NGF or other neurotrophins, is unresolved: serum NGF levels appear unaffected by aging (115, 116) or modestly decrease (117), as does serum BDNF levels (118). While *Ngf* expression in bone is mechanically regulated, and is induced less in aged mice compared to younger mice (119), if attenuated load-induced expression with increasing age impacts sympathetic or sensory signaling in the skeleton requires greater elaboration. Further, detailed studies defining the contribution of NGF to post-menopausal vs. sex-independent involutional bone loss are lacking.

## Conclusions

Extensive and sustained efforts reveal that the skeleton is richly innervated by sensory and sympathetic nerves which appear during and participate in skeletal development; further investigations have implicated these same nerve fibers in skeletal homeostasis and adaptation, as well as contributions toward bone loss with age. Yet, with each discovery, the relationships become more complex, demanding more precise interrogation and articulation in order to weave together a precise narrative. Indeed, the development of this narrative is hampered by a variety of questions. To what extent conclusions about the impact of sympathetic or sensory fiber number, density, etc. on the skeleton limited by an experimental approach that may not be as robust as assumed. For example, the decalcification of bone that is necessary for its immunohistological evaluation can prevent retention of neurologic markers, as demonstrated in (74), wherein labeling of TrkA, p75, and NGF in the periosteum and bone marrow was diminished in specimens that had undergone decalcification. Observations such as these motivate the opportunity to utilize or develop models whose results are less ambiguous and with greater fidelity, such as cell-specific fluorescent reporter mice. Furthermore, despite the mandate from the National Institutes of Health to include sex as a biological variable, many of the studies reviewed here used animals of a single sex. Provided the overwhelming fact of skeletal sexual dimorphism and evidence supporting sexual dimorphism in neurotrophin and receptor expression (120–122), the opportunity to establish correlation, if not causation, is missed. Indeed, a novel role for kisspeptin-expressing cells within the arcuate nucleus—wherein estrogen receptor alpha drives central and peripheral energy metabolism to exert inhibitory effects on bone mass—was discovered recently in female, but not male, mice (123). Studies like these, and other reports whose seeming contradictions with previous reports may originate in sexual dimorphism, reveal the obligation to evaluate both sexes. Furthermore, this study also illuminates another area of nerve-bone interaction outside scope of the present review—signaling in the central nervous system. In total, more research to resolve outstanding issues and improve our knowledge of nerve-bone interaction may permit the use of these signaling mechanisms to combat skeletal diseases, effectively treat skeletal pain, increase bone mass in healthy individuals, and address age-related declines in skeletal health.

## Author Contributions

All authors contributed to development of this article, reviewed pertinent literature, wrote, and edited the manuscript.

## Conflict of Interest

The authors declare the absence of any commercial or financial relationships that could be construed as a potential conflict of interest.
